# Is religion opium for the masses? Role of spirituality in mental health

**DOI:** 10.1192/j.eurpsy.2025.1290

**Published:** 2025-08-26

**Authors:** P. Główczyński, P. Dębski, K. Badura-Brzoza

**Affiliations:** 1 Clinical Department of Psychiatry, Faculty of Medical Sciences, Medical University of Silesia, Tarnowskie Góry, Poland

## Abstract

**Introduction:**

The relationship between human mental health and religiosity (or broadly understood spirituality) has been the subject of interest of scientists, including philosophers, for hundreds of years. For centuries, religion has played a key role in shaping people’s psychosocial and moral development, both positively and negatively. Spirituality should also not be treated as a practice solely related to attending temples or praying. Certainly, a common feature of all types of spirituality is the search for a deeper meaning that goes beyond the material experience of life.

**Objectives:**

The aim of the study was to analyze available scientific research in terms of the relationship between religiosity, spiritual practices and faith with selected parameters of human mental health.

**Methods:**

The PubMed and Cochrane Library databases were searched for in this analysis. The search phrases used were: “spirituality/religion AND mental health”, “spirituality/religion AND depression”, “spirituality/religion AND anxiety”, “spirituality/religion AND suicide” [Image 1].

**Results:**

The relationship between spiritual practices or religiosity and the occurrence of depressive symptoms is not clear. By far the largest number of analyses was conducted in the United States, where, due to the heterogeneity of society, different beliefs and spirituality are present. Despite the high heterogeneity of studies, a high level of spirituality is usually associated with a lower intensity of anxiety symptoms. Obsessive thoughts are relatively often associated with religious motives. An interesting assumption is the concept that religion as a set of rules and practices that require strict adherence may be the foundation for the development of OCD. Religion may reduce the frequency of fantasizing about death, which is confirmed by studies conducted in various religious populations. Differentiating psychotic symptoms from spiritual experiences may be a significant diagnostic difficulty.

**Image 1:**

**Figure:**
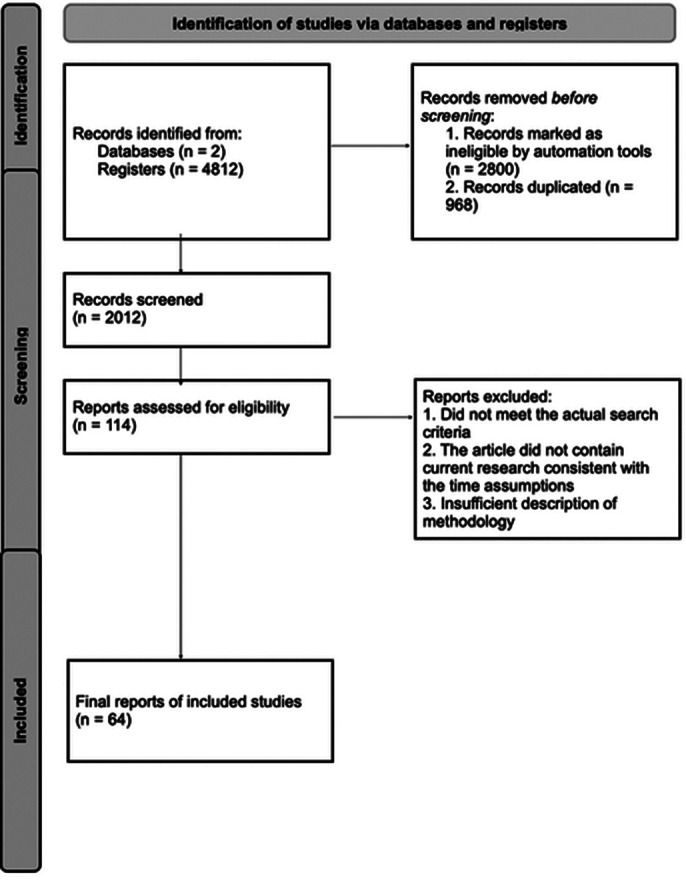

**Conclusions:**

The intensive period of socio-cultural and economic changes has an impact on people’s attitudes towards religiosity, their sense of belonging to a religious group, or spirituality in general. As the available analyses show, spirituality can be a protective or burdensome factor, or it can have no impact on a person’s mental well-being. The studies available in medical databases show significant heterogeneity in this respect, which makes their direct comparison difficult. Another methodological problem seems to be the assessment of spirituality. We cannot forget about the cultural context in the assessment of societies. An interesting analysis would be to assess the relationship between specific forms of spiritual practice and their intensity with reference to tradition, gender, age, and geographical region. Considering the aspect of spirituality in human life is part of a holistic view in medicine.

**Disclosure of Interest:**

None Declared

